# Long head of the biceps intra-articular tenotomy using needle arthroscopy under local anesthesia: preliminary results and technical note

**DOI:** 10.1186/s40634-022-00508-5

**Published:** 2022-07-22

**Authors:** Claudio Rosso, Kushtrim Grezda, Philipp R. Heuberer

**Affiliations:** 1 Shoulder and Elbow Center, ARTHRO Medics, Thannerstrasse 45, 4054 Basel, Switzerland; 2 University of Basel, Basel, Switzerland; 3 Department of Orthopaedics and Traumatology, University Hospital Basel, Basel, Switzerland; 4 Schulterzentrum.wien, Vienna, Austria; 5 Healthpi Medical Center, Vienna, Austria; 6 AURROM - Austria Research Group for Regenerative and Orthopedic Medicine, Vienna, Austria

## Abstract

**Purpose:**

Arthroscopic isolated biceps tenotomy is a procedure successfully performed in patients with degenerative rotator cuff tears which offers good clinical results. With this article, we describe the technique of biceps tenotomy with needle arthroscopy in local anesthesia and the results of first patients treated from 2018.

**Methods:**

Thirteen patients with irreparable rotator cuff tear treated with arthroscopic needle biceps tenotomy under local anesthesia were included. Constant score and active/passive flexion were recorded preoperatively and at 3 months postoperatively.

**Results:**

The average age of the patients was 71 ± 7 year old. All the patients were available for the follow-up. The Constant score significantly improved from pre- to postoperatively (44 ± 8.9 to 63.1 ± 14.2, *p* < 0.05). Active flexion improved from preoperatively 115 ± 24° to 145 ± 31° postoperatively (*p* < 0.05), while passive flexion did not significantly improve.

**Conclusion:**

This technique can be safely performed in the elderly patient with irreparable rotator cuff tears and pain refractory to conservative measures.

**Supplementary Information:**

The online version contains supplementary material available at 10.1186/s40634-022-00508-5.

## Introduction

Needle arthroscopy is an increasingly applied technique that is well established in the evaluation of knee pathologies. The main advantage is that it can be performed under local anesthesia in patients for whom general anesthesia is not convenient; moreover, this procedure can be conducted in an outpatient clinic setting. Until recently, only diagnostic arthroscopy has been performed [1]. Recently, surgeons have developed interventions in the knee, but in shoulder surgery, needle arthroscopy has not yet made a great breakthrough [2]. Needle arthroscopy in the shoulder can be used for second-look surgery, with minimal material needed and a minimal setup [3]. Gauci et al. recently published a feasibility study of biceps tenotomy with needle arthroscopy in a cadaveric setting [4].

Elderly and multimorbid patients with biceps tendinopathy usually accompanied by cuff tear arthropathy may no longer respond to repetitive corticosteroid injections [5]. However, due to comorbidities, such patients are either not able or not willing to undergo surgery under general anesthesia.

To our knowledge, our group performed the first biceps tenotomy under local anesthesia using a needle arthroscope in 2018. Our goal was not only to use needle arthroscopy for diagnostic tools but also to successfully perform biceps tenotomy with needle arthroscopy in elderly patients under local anesthesia. In this article, the materials needed, the technique, functional results and “tips and tricks” are described.

## Material and methods

From 2018 to 2021, arthroscopic needle biceps tenotomy was performed in 13 patients. The surgeries were performed by two surgeons who authored this manuscript (C.R. and Ph.H.), and the surgeries took place in two different centers. The first 3 surgeries were performed in the OR room, while 10 others were performed in the outpatient clinic. The inclusion criteria were as follows: patients with symptomatic degenerative rotator cuff tears diagnosed with Arthro-MRI; failure of extensive conservative treatment such as physical therapy and repetitive cortisone injections for at least 6 months; irreparable rotator cuff tears (retraction type [Patte III] and fatty infiltration [Goutallier 3 or 4]); patients older than 65 years; and multimorbid patients unwilling or unable to undergo general anesthesia. Before the procedure, all the patients were asked to tell the surgeon if they felt unwell or were uncomfortable during the procedure, and since the surgeries were performed under local anesthesia, direct communication with the patients was maintained during the whole procedure. All patients were treated under local anesthesia (ropivacaine 0.75%) with either Mi-Eye (Trice Medical, Malvern, PA, USA) or NanoScope (Arthrex, Naples, FL, USA). Patients were surveilled for approximately 30 min after the procedure and then discharged from the hospital. Full range of motion of the shoulder and elbow was immediately allowed, as in standard biceps tenotomy. The procedure time was measured in minutes. Patients were followed for a minimum of 3 months. Absolute constant scores with subscores, active/passive flexion and the “Popeye sign” were recorded preoperatively and 3 months postoperatively.

## Statistical analysis

Statistical analysis was carried out using IBM SPSS Statistics Version 24 (Armonk, New York, USA). Univariate analysis was performed using Student’s t test. The significance level was set to *p* < 0.05.

## Surgical technique

### Setup material

**Fig. 1:**
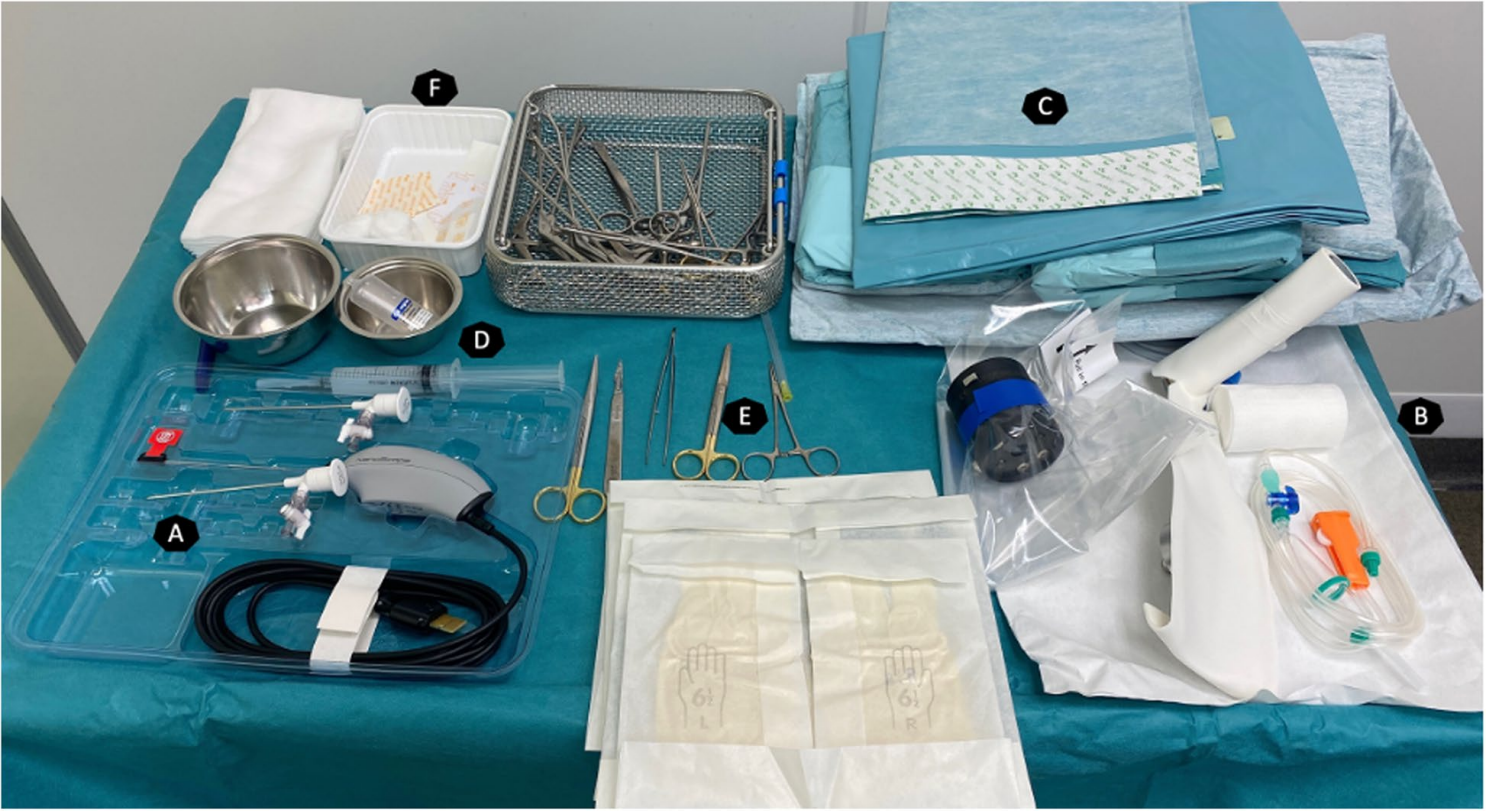
Sterile setup material. NanoScope (**A**) connection kit for the compression cuff (**B**) sterile draping (**C**) ropivacaine and syringe (**D**) basic surgical instruments (**E**) sterile dressing (**F**)

As seen in Fig. 1, the following are needed:

- disinfection material
- shoulder draping
- needle arthroscope (MiEye2, Trice Medical, Malvern, PA, USA) or NanoScope (Arthrex, Naples, FL, USA)
- 10 ml of local anesthetic ropivacaine 0.75%
- No. 11 blade
- Marker pen
- 2 L Ringer’s lactate
- Compression cuff

**Fig. 2:**
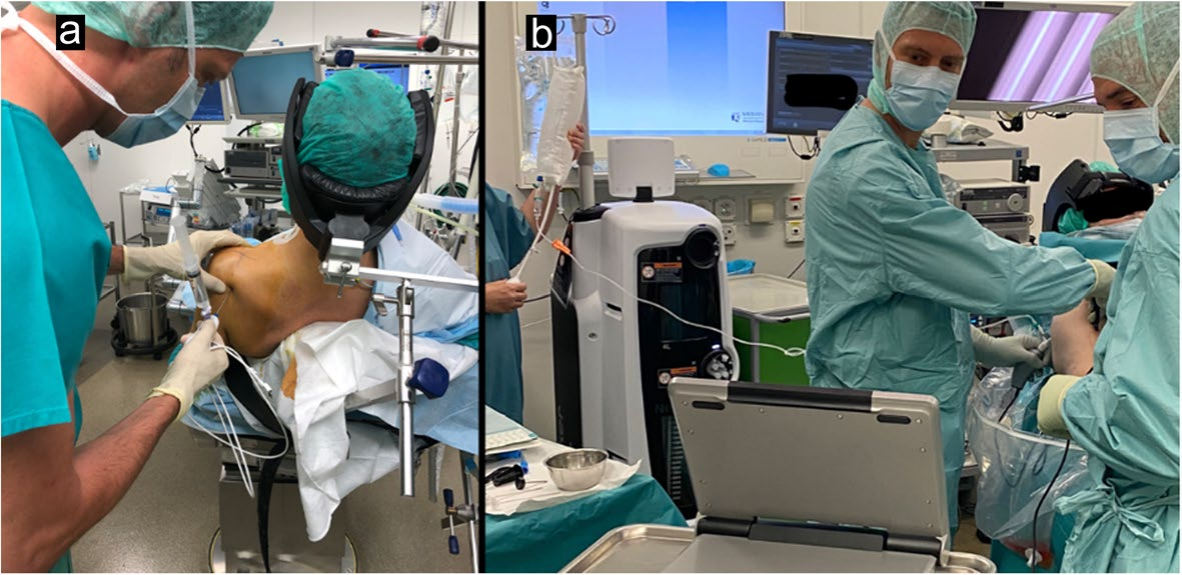
**a**) Needle arthroscope with a syringe; **b**) compression cuff during surgery

Initially, the shoulder was insufflated with saline with a syringe attached to the needle arthroscope (Fig. 2A). Then, a compression cuff for the Ringer`s lactate was used as an optimal way to obtain a good view (Fig. 2B).

### Patient setup

- As per the protocol of the hospital in pure arthroscopies without foreign bodies, such as inserted anchors: no administration of any preoperative antibiotics.
- Beach chair position or lateral decubitus
- Standard disinfection from the hand to the neck of the patient
- Partial draping of the shoulder, from the hand until above the elbow in the distal part and a circumferential drape above the shoulder, where the arm passes through.

### Procedure

**Fig. 3:**
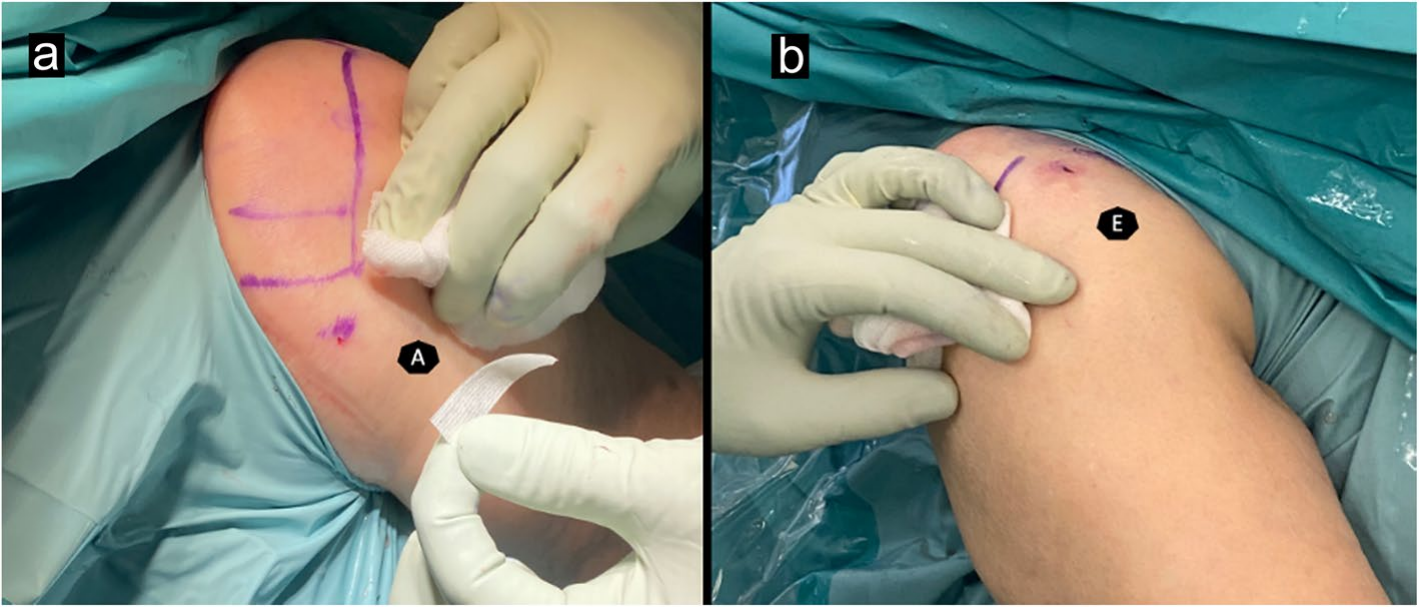
Standard portals. **a**) Standard posterior portal (A); **b**) anterior rotator interval portal (E)

1. After the patient is disinfected and draped, the following portals are marked with a marker pen: standard posterior (A) portal and anterior rotator interval portal (E) (Fig. 3). First, the skin is continuously infiltrated with local anesthetic from the (A) portal pointing toward the coracoid until the needle is advanced into the joint. Second, the (E) portal is infiltrated from the skin (intracutaneously) to the glenohumeral joint.
2. The standard posterior (A) portal is incised with the no. 11 scalpel, creating a 1–2 mm incision. Now, the needle arthroscope is introduced pointing toward the coracoid as in usual 4 mm arthroscopy. The water flow from the compressed Ringer`s lactate is connected and established. An intraarticular view is verified.
3. The anterior rotator interval (E) portal is incised with the no. 11 scalpel, creating a 1–2 mm incision, and the instrument is introduced. Optimally, a 2.7 mm cannula system is used to ease the change of instruments (arthroscopic mini-scissors and mini-knife), which come with the NanoScope set.
4. A short diagnostic arthroscopy should be performed while being aware that the scope has a 0° optic so that the surgeon is looking straight in line with the needle. One should also be aware that the 1.9 mm scope does not allow for manipulations, as it will bend.
5. The long head of the biceps tendon (LHB) insertion is identified with the needle arthroscope, and the nanoarthroscopic scissor that is a part of the set is introduced into the (E) portal.
6. The LHB is tenotomized, and tenotomy is verified under direct visualization.
7. A “Popeye” sign of the upper arm is a clear sign that the needle arthroscopy LHB tenotomy was successful.
8. Optionally, an intraarticular corticosteroid injection is administered to reduce postoperative inflammation.
9. The wounds are closed with wound closure strips, and the shoulder is bandaged.

## Results

All patients were available at the 3-month timepoint for follow-up, and this was the last follow-up for all the patients. The average age of the patients at the time of surgery was 71 ± 7 years (mean ± SD). The average procedure time was 5 min.

In the final postoperative follow-up objective clinical examination, the “Popeye” sign was apparent in 9 patients (69.2%). Constant scores, except for the strength subscore, were improved significantly at 3 months (44 ± 8.9 to 63.1 ± 14.2, *p* < 0.05). Active flexion significantly improved from 115 ± 24 to 145 ± 31° (mean ± SD, *p* < 0.05), while there was no significant difference between passive flexion values. Outcomes are summarized in Table 1.

**Table 1:** Functional scores

Variables	Preoperative	Postoperative	*P* value
Constant scores			
Pain	3.2 ± 2.8	10.8 ± 3.5	< 0.05
Activity	8.2 ± 2.7	14.7 ± 4.1	< 0.05
Mobility	27.5 ± 7.8	31.7 ± 7.9	< 0.05
Strength	5.1 ± 2.8	5.9 ± 2.8	n.s.^a^
Absolute total score	44 ± 8.9	63.1 ± 14.2	< 0.05
Range of motion			
Active flexion	115 ± 24	145 ± 31	< 0.05
Passive flexion	163 ± 45	171 ± 19	n.s.*

Scores at the 3-month follow-up

^a^*ns* not significant

## Discussion

The most important finding was that biceps tenotomy in local anesthetic by means of needle arthroscopy is feasible and yields good postoperative outcomes. Constant scores and subscores (especially pain) significantly improved with this simple procedure in the group of patients who could not receive general anesthesia or who were willing to undergo a fast outpatient procedure. In addition to the Constant score, active flexion significantly improved, as reported by other authors [6], which allowed patients to have a greater range of motion and ultimately meet their expectation from the intervention. The findings of this study are in line with those in other articles reporting isolated biceps tenotomy under general anesthesia using a 4 mm arthroscope with satisfactory results [4, 5, 7]. While Gauci [4] showed that biceps tenotomy with needle arthroscopy is technically feasible and Veen [7] found that isolated long head of biceps tenotomy in patients with degenerative rotator cuff tears yields good mid-term clinical results, we believe that this is the first study to demonstrate the feasibility of and good results from needle arthroscopy biceps tenotomy in local anesthesia.

In recent years, diagnostic shoulder needle arthroscopy has emerged as a procedure that provides a fast track with a short operative time and greater efficiency [1, 2, 4], which makes it an appealing solution for elderly patients with indications for shoulder arthroscopy in whom general anesthesia is contraindicated and other conservative measures have failed. Needle arthroscopy has proven to be successful for diagnostic purposes in the shoulder [1–3] and, lately, for other more invasive procedures, such as anterior labrum repair [8] and rotator cuff repair [9], under general anesthesia. Recently, needle arthroscopy has started to be performed safely in narrower joints, such as the ankle [10, 11], where the conventional scope can expose the patient to unnecessary iatrogenic damage.

In our patient series, we were able to perform biceps tenotomy in a short period of time (approximately 5 min), by using a local anesthetic with little chondrotoxicity, such as ropivacaine 0.75% [4], and patients felt comfortable during the whole procedure. Therefore, the authors felt that they were able to offer the patients a safe and successful procedure by avoiding general anesthesia and tenotomizing the biceps tendon as would be done in general anesthesia. We believe that this procedure is safe in patients with comorbidities (ASA IV) and has the potential to be safely repeated for other pathologies of the shoulder joint, including but not limited to biopsy, loose body removal and second-look arthroscopy.

In addition to several advantages, needle arthroscopy also has various disadvantages mainly due to the 0-degree view, which may be a potential difficulty for surgeons that have used only a 30- or 70-degree scope. This disadvantage can be avoided by practice in the laboratory setting and by performing several shoulder needle arthroscopies for diagnostic purposes before progressing to more invasive procedures. Another limitation is that manipulation with the scope should be carried out with diligence due to the risk of device bending. This can be avoided by placing the portals precisely perpendicular to the biceps tendon, which we believe to be the cornerstone of a successful procedure. Moreover, needle arthroscopes are meant to be used with a syringe. However, this is not feasible in the shoulder since the volume in the shoulder is much higher than that in a knee. However, this issue was addressed by using a compression cuff for the Ringer`s lactate during the procedure. Swelling of the shoulder joint is not an issue with this short procedure that takes a maximum of 15 min. Currently, we do not see needle arthroscopy as a good option for young patients since the potential for iatrogenic cartilage damage is higher due to the learning curve.

## Conclusion

In conclusion, biceps tenotomy with needle arthroscopy under local anesthesia is a feasible and reliable method that can be conducted safely by surgeons who regularly perform conventional arthroscopy. We recommend the procedure for selected elderly patients with biceps tendinopathy in whom general anesthesia is contraindicated due to multiple comorbidities.

## Supplementary Information

**Additional file 1.**
